# The Influences of Soybean Agglutinin and Functional Oligosaccharides on the Intestinal Tract of Monogastric Animals

**DOI:** 10.3390/ijms19020554

**Published:** 2018-02-12

**Authors:** Li Pan, Mohammed Hamdy Farouk, Guixin Qin, Yuan Zhao, Nan Bao

**Affiliations:** 1 Key Laboratory of Animal Production, Product Quality and Security, Ministry of Education, Key Laboratory of Animal Nutrition and Feed Science, Jilin Province, College of Animal Science and Technology, Jilin Agricultural University, Changchun 130118, China; panli0628@126.com (L.P.); zhaoyuan4CL52@126.com (Y.Z.); baonan203@163.com (N.B.); 2 Animal Production Department, Faculty of Agriculture, Al-Azhar University, Nasr City, Cairo 11884, Egypt

**Keywords:** soybean agglutinin, oligosaccharides, intestinal health, intestinal structure, microflora

## Abstract

Soybean agglutinin (SBA) is a non-fiber carbohydrate-related protein and the main anti-nutritional factor that exists in soybean or soybean products. SBA possesses a specific binding affinity for *N*-glyphthalide-d-galactosamine or galactose and has a covalently linked oligosaccharide chain. SBA mediates negative effects on animal intestinal health by influencing the intestinal structure, barrier function, mucosal immune system, and the balance of the intestinal flora. Functional oligosaccharides are non-digestible dietary oligosaccharides that are commonly applied as prebiotics since the biological effects of the functional oligosaccharides are to increase the host health by improving mucosal structure and function, protecting the integrity of the intestinal structure, modulating immunity, and balancing the gastrointestinal microbiota. The purpose of this review is to describe the structure and anti-nutritional functions of SBA, summarize the influence of SBA and functional oligosaccharides on the intestinal tract of monogastric animals, and emphasize the relationship between SBA and oligosaccharides. This review provides perspectives on applying functional oligosaccharides for alleviating the anti-nutritional effects of SBA on the intestinal tract.

## 1. Introduction

Soybean agglutinin (SBA) is a major anti-nutritional factor that represents 5–7% of the soybean. This anti-nutritional factor is a glycoprotein that specifically binds *N*-acetylgalactosamine or galactose and each subunit of SBA has a covalently linked oligosaccharide chain. SBA has a typical four-polymer structure of leguminous plant lectin but is more stable than the other legume lectins due to a large number of hydrogen bonds and hydrophobic interactions between the two monomers of the SBA molecule [1]. Although the biological activity of SBA can be reduced by proper heating, a considerable quantity is still found. This residual SBA can affect intestinal health by influencing intestinal structure [2], barrier function [3,4], the mucosal immune system [5], and the balance of the intestinal flora [6]. The anti-nutritional effects of SBA on animals differ depending on the animal species and age and lectin dosage. For example, Grant et al. [7] reported that the anti-nutritional effect of SBA on monogastric animals is greater than on ruminants. This may be related to the rumen fermentation of the ruminant, which reduces the activity of the anti-nutritional factors.

Functional oligosaccharides are found widely in plants, algae, bacteria, and higher fungi [8]. Such oligosaccharides cannot be digested in the gastrointestinal tract. Therefore, they can be named as non-digestible dietary oligosaccharides or non-nutrient oligosaccharides. However, functional oligosaccharides can be fermented in the large intestine by indigenous bacteria and are preferably used by probiotic bacteria [9]. Therefore, they are commonly applied for many purposes, such as prebiotics, nutrients, feeds, pharmaceuticals, cosmetics, and immunostimulating agents [10]. Moreover, these oligosaccharides have been found to be effective for the host’s well-being [11] by protecting the integrity of the intestinal structure, enhancing immunity, facilitating mineral absorption, improving gastrointestinal normal flora proliferation, and suppressing pathogens [12]. 

Each subunit of SBA has a covalently linked oligosaccharide chain and contains *N*-acetylgalactosamine and mannose in its structure. In addition, SBA has a negative effect on intestinal structure and function, while some functional oligosaccharides can improve intestinal health. According to the above conclusions, we hypothesized that SBA and functional oligosaccharides have some direct and indirect antagonistic actions on the intestinal system. Therefore, this review mainly focuses on understanding the structure and anti-nutritional functions of SBA, summarizes the influences of SBA and functional oligosaccharides on the intestinal tract of monogastric animals, and emphasizes the relationship between SBA and oligosaccharides. Finally, the herein paper shows the mitigating effects of functional oligosaccharides in the intestinal damage caused by SBA.

## 2. Soybean Agglutinin

Soybean agglutinin (SBA) is a family of similar legume lectins. This dietary non-fiber carbohydrate-related protein is the only bioactive molecule with a high content in soybean or soybean products and is one of the main anti-nutritional factors that affect the quality of soybean. The understanding of SBA begins with the discovery of its agglutination activity since one of the common features of this kind of substance is its ability of hemagglutination [13]. The change in the hemagglutinin structure leads to an increase in body weight of animals fed processed soybean [14]. 

SBA is one kind of soybean anti-nutritional factor and is more stable than the other legume lectins. Thus, SBA can resist degradation by proteases under in vitro and in vivo conditions. The undigested SBA then binds to the surface of intestinal epithelial cells and induces deleterious effects, depending on its formations. Commission Implementing Regulation (UE) 2016/1833 concerns the authorization of bean lectin preparations (*Phaseolus vulgaris* lectin) as a feed additive for suckling piglets in order to prevent the negative effects during weaning.

SBA—despite its similarity to antibodies due to the fact that it is able to combine with appropriate antigens—does not constitute an element of the immune system. Its structure is different and its specificity is limited to carbohydrates.

### 2.1. Soybean Agglutinin (SBA) Structure

SBA possesses a specific binding affinity for *N*-glyphthalide-d-galactosamine or galactose and has a typical quaternary structure. The molecular weight of SBA is 120 kDa. SBA consists of four similar subunits that each have a molecular weight of 30 kDa. Each subunit of SBA contains a tightly bound Ca^2+^ and Mn^2+^, which are necessary for the glucose binding activity of SBA. The subunit also has a covalently linked oligosaccharide chain containing nine mannose and two *N*-glyphthalide-glucosamines (Man_9_GlcNAc_2_). Each subunit consists of a flat region consisting of six strands of beta-folding, a bended zone of seven strands of β-folds, and five arc regions. A complete SBA molecule contains about 4.5% d-mannose and 1.5% *N*-phthalide-glucosamine. The oligosaccharide chains inherent in SBA have two configurations: one 3,6 branch point configuration and two 3,6 branching point configurations. 

SBA can be considered as “dimers of dimers”. SBA is more stable than the other legume lectins, and needs more energy to form tetramer from monomers [1]. The main reason is that there is a large number of hydrogen bonds and hydrophobic interactions between the two monomers of the SBA molecule [15]. 

Dessen et al. [16] pointed out that a C-terminated subunit (240 AA) and a non-truncated subunit (253 AA) are associated with each other in the SBA four subunits. Each subunit contains 270 amino acid residues. SBA molecules have a low content of cysteine and methionine amino acids but possess a rich content of hydroxyl amino acids, especially 4-hydroxyproline content that is higher [17].

SBA is a non-fiber carbohydrate-related protein that precipitates complex carbohydrates or polysaccharide agglutinate cells [18]. The binding activity of SBA for sugar does not depend on the inherent sugar chain of SBA but on the active sites in the peptide chain of SBA, which are the specific sugar binding sites. In addition, the specific binding of SBA to oligosaccharides is related to the conservative amino acid residues in the binding region of SBA [19]. The above characteristics lead to a decrease in the potential bioactivity of feedstuffs.

### 2.2. SBA Anti-Nutritional Functions

**Figure 1:**
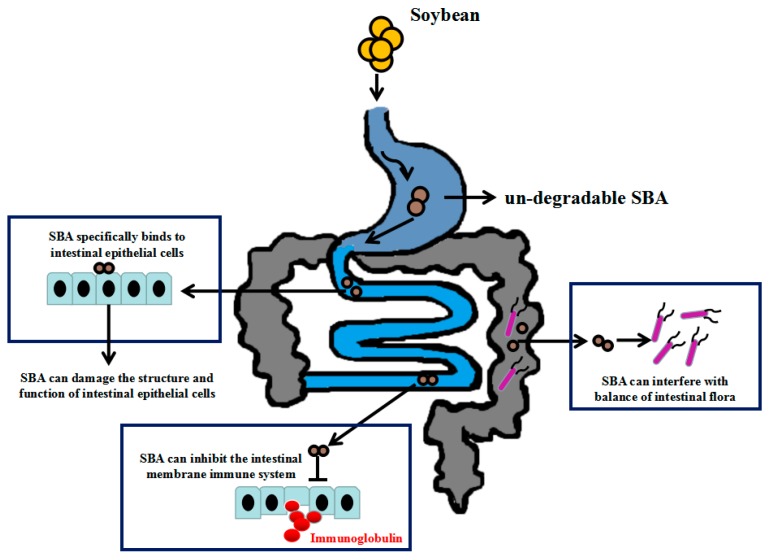
Anti-nutritional functions of soybean agglutinin (SBA).

Due to the stability of the SBA structure, SBA can resist degradation by proteases in vitro and in the gastrointestinal tract. Such undegradable SBA can specifically bind to intestinal epithelial cells. The specific binding is a prerequisite and necessary condition for its anti-nutritional effect. After combining with the intestinal surface, SBA can be internalized by the epithelial cells to enter the blood flow, leading to a wide range of systemic anti-nutritional effects as shown in Figure 1.

#### 2.2.1. SBA and Intestinal Morphological and Structural Patterns

SBA molecules are able to alter intestinal morphology [2]. The addition of low SBA levels (1.2 mg SBA per gram of diet) may contribute to the growth of the rat small intestine [20], while in a high level (2.0 mg/g) it damages the intestinal morphology. For example, the intestinal epithelial cells of piglets treated with 0.5 or 2.0 mg/mL SBA lead to cellular morphological changes (boundaries between adjacent cells were ambiguous) and cellular growth patterns [3,4]. The exact mechanism of this ambiguous pattern is still unclear.

Feeding of raw soybean leads to reductions in both feed conversion ratios and growth performance of rats since SBA increases cellular hyperplasia and hypertrophy of the small intestine [21]. 

Binding of SBA by mammalian intestinal epithelial cells has deleterious effects in vivo [7] and in vitro [22]. In Atlantic salmon, SBA probably exerts its anti-nutritional functions by binding to the intestinal brush border membranes [23]. Dietary full-fat soybean meals increase the number of goblet cells, decrease the absorptive vacuoles in enterocytes, increase the cellularity of the lamina propria, shorten the microvilli, and increase the formation of microvillar vesicles in Atlantic salmon (*Salmo salar*). Such morphological changes in the distal intestine may be related to the anti-nutritional factors in soybean such as SBA or protease inhibitors [24]. In addition, SBA specifically recognizes chicken intestinal epithelial cell (IEC) membranes and SBA can be internalized by pinocytosis. This process alters cellular viability, apical membrane transport mechanisms, microvilli development, and brush border enzyme expression [25].

SBA disrupts the intestinal brush border membrane [26], resulting in activity inhibition of various enzymes in the brushing edge of the small intestine [27]. When SBA is combined with the surface of the inner cavity of the small intestine, it destroys the function and structure of the brush border membrane and some related enzymes [28], which seriously affect the digestion and absorption of nutrients [29]. A dose of 2.7 μM SBA can non-competitively inhibit the activity of intestinal enterokinase in the brush edge of the rat duodenum [30]. Similar trends with doses of 0.024% and 0.048% SBA in the diet significantly reduce the activity of maltase and invertase in the brush edge membrane of the duodenum, jejunum, and ileum [2].

#### 2.2.2. SBA and Intestinal Mechanical Barrier Function

The intestines normally exhibit some permeability, which allows nutrients to pass through the gut while maintaining barrier function to keep potentially harmful substances from leaving the intestine and migrating to the body more widely [31]. 

The binding of SBA induces the synthesis of stress-induced proteins that leads to the reestablishing of cell-to-cell relationships during monolayer reorganization following injury in rat corneal endothelium [32]. SBA increases membrane permeability, inhibits cell viability, and reduces the levels of tight junction proteins (occludin and claudin-3), leading to a decrease in mechanical barrier function in intestinal epithelial cells [3,4]. Zhao et al. [33] also indicated that high-dose SBA (0.1–0.2% in diet) can increase the intestinal permeability and damage the intestinal barrier function by reducing intestinal epithelial tight junction protein occludin or zonula occludens-1 protein (ZO-1) expression in piglets; however, low doses of SBA (0.05% of total diet) have no effects. Thus, SBA is able to affect the protective patterns of the intestinal tract.

#### 2.2.3. SBA and the Intestinal Mucosal Immune System

The intestinal digestive tract contains diffuse lymphoid tissue, solitary lymph nodules, aggregated lymphatic nodules, lymphocytes, macrophages, and plasma cells. Such immune cells produce many secretory immunoglobulins to protect the integrity of the cell membrane and the health of the body and participate in the first line of defense for the body’s immune defense [34]. When the intestinal mucosal immune system is affected by an antigen, the lymphoid tissue in the mucosa immediately produces an immune response and secretes immunoglobulins into the digestive tract to resist the invasion of bacteria, viruses, and other harmful antigens from the digestive tract [35]. 

SBA is able to induce a local inflammatory reaction. SBA increases bone marrow cell proliferation, stimulates monocyte/macrophage colony growth [36], and marks the late stages of macrophage differentiation [37]. In a local injection, SBA contributes its harmful effects on the small intestine by increasing the population of mononuclear cells, the numbers of CD4^+^/CD8^−^ lymphocytes, the expression of CD11/CD18 surface molecules, and the number of circulating neutrophils and by inhibiting neutrophil migration in rats. Also, when SBA is present in the blood circulation, an inhibitory effect on neutrophil migration is observed, suggesting an anti-inflammatory effect [38].

SBA can also affect mucosal immune systems. Greer and Pusztai [5] reported that continuous feeding of SBA leads to a decreased immunological response in rats by inhibiting the intestinal mucosal immune system, which could not produce enough secretory immunoglobulins to prevent the absorption of SBA. Degranulation of mast cells (a type of white blood cell) is directly triggered by the uptake of SBA by epithelial cells, increasing histamine secretion. These biological processes lead to increased vascular permeability and serum albumin loss. These negative effects of SBA could be extended to the gut’s microbial composition.

#### 2.2.4. SBA and the Balance of Intestinal Flora

The gastrointestinal tract contains thousands of different microbial species. Such species play an important role in the immune system and several other functions [39]. For example, gastrointestinal microbiota is an important participant in human metabolism. It provides substrates, enzymes, and energy for human metabolic processes. At the same time, the fatty acids produced by metabolism can promote the growth and differentiation of human epithelial cells and can participate in the synthesis of vitamins and the absorption of all kinds of ions [40]. In recent years, the relationship between intestinal microorganisms and obesity has been widely investigated. Some researchers found that *Firmicutes* and *Bacteroidetes* are related to obesity [41]. In addition, there is also a close relationship between intestinal microbial species and diabetes. Some differences in the intestinal microbial composition have been found between diabetic patients and non-diabetic patients, showing a lack of butyric acid bacteria in diabetics [39]. 

SBA can interfere with the balance of intestinal flora [6]. Diets supplemented with purified SBA increase the content of volatile fatty acids in the ileal digesta of piglets, indicating enhancement of the intestinal microbial activity [42]. When soybean meal is used to replace 75% of the defatted fishmeal, significant differences can be tested in the intestinal microbiota composition. Such treatment lowers the abundance of *Firmicutes* in contrast to *Proteobacteria*, *Bacteroidetes*, and *Planctomycetes*. At the genus level, SBA can significantly lower the abundance of *Lactococcus*, *Geobacillus*, *Pseudomonas*, *Streptococcus*, *Bacillus*, and *Acinetobacter*. Conversely, SBA enhances the abundance of *Cetobacterium*, *Planctomyces*, *Shewanella*, *Thermomonas*, *Rubrivivax*, and *Carnobacterium*. Therefore, SBA is considered as the main cause of these changes [43]. 

The mechanisms by which SBA affect intestinal flora may have different aspects: first, binding of SBA to the small intestinal epithelial cells alters the glycan structures of the small intestinal mucosa and changes the attachment sites for some bacteria on the intestinal surface, thereby selectively stimulating the transient growth of some bacteria; second, the induction of SBA provides abundant nutrition for bacteria (such as from the loss of serum protein and increased loss of intestinal cells); third, SBA destroys the intestinal mucosal immune system, reducing the secretion of secreted ImmunoglobulinA (IgA), which inhibits bacterial proliferation [39]. Thus, SBA has three possible mechanisms to increase the microbial activity.

Therefore, SBA possesses different negative effects on the intestinal structure, mechanical barrier function, mucosal immune system, and intestinal flora. Finally, SBA can reduce feed utilization, growth performance, disease, and even mortality. Despite the above deleterious effects of SBA, there are some carbohydrates that possess antagonistic biological roles.

### 2.3. The Positive Effects of SBA

The beneficial bioactive effects of SBA have been investigated. Some positive effects, including antitumor, antifungal, antiviral, and antibacterial activities, have also been found for SBA [44]. For example, Panda et al. [45] studied the antitumor effects of SBA and indicated SBA-mediated autophagy, apoptosis, and DNA damage in the Henrietta Lacks (HeLa) strain of cancer cells. In addition, SBA is involved in the generation of reactive oxygen species (ROS) in a dose-dependent manner.

According to the structure and positive functions of SBA, this protein has been widely applied in medical research. For example, SBA binding has been investigated as a useful tool for detecting stomach cancer [46] and could be conjugated to silver nanoparticles for treating breast cancer [47]. 

## 3. Functional Oligosaccharides

Oligosaccharides are saccharide polymers containing a small number (typically three to ten) of monosaccharides (simple sugars) [48]. Oligosaccharides (saccharide polymers) are normally present as glycans. Such polymers provide beneficial effects on intestinal health and the immune system, such as immunomodulatory activity, anticancer activity, and complement activation. These oligosaccharides are widely found in plants, algae, bacteria, and higher fungi [8]. 

Oligosaccharides can be divided into common oligosaccharides and functional oligosaccharides. The first type of oligosaccharides (including sucrose, lactose, and maltose) mainly provides energy and pleasant sweetness for the body but has no effect on the growth of probiotic bacteria in the intestines. Functional oligosaccharides can have special physiological functions in humans, monogastric animals, and plants. These organisms have no enzyme systems in the monogastric intestinal tract to hydrolyze such molecules [49]. Therefore, such oligosaccharides can also be named as non-digestible dietary oligosaccharides or non-nutrient oligosaccharides. 

However, functional oligosaccharides can be fermented in the large intestine by indigenous bacteria and are preferred by probiotic bacteria [9]. Thus, such saccharides are commonly applied for many purposes, such as prebiotics, nutrients, feeds, pharmaceuticals, cosmetics, and immunostimulating agents [10]. Many oligosaccharides are considered as prebiotics [50]. The prebiotics are non-digestible molecules and therefore are resistant to digestion and absorption. Hence, they provide a low energy source and instead are broken down by the enteric microbiome, supporting specific bacteria such as *Bifidobacteria* and *Lactobacilli* [51,52].

Moreover, functional oligosaccharides can have a positive effect on different well-being aspects of the host, such as immune modulation, intestinal health, gastric microbial function, calcium absorption, and bone mineral density, particularly among adolescents [53]. The increased absorption is attributed to an increase in mineral availability and to the binding/sequestering capacity of non-dietary oligosaccharides as the intestinal pH decreases as a result of oligosaccharide utilization by microorganisms in the intestine [54].

Below are different types of functional oligosaccharides, sharing similar and typical functions. 

### 3.1. Galacto-Oligosaccharides

Galacto-oligosaccharides are non-digestible dietary carbohydrates with short galactosyl chain units that are produced by lactose fermentation and can be commercially produced from lactose using the galactosyltransferase activity of b-galactosidase [55]. Owing to the structural and functional characteristics, such saccharides can be defined as prebiotics. While they resist the gastric acidity and hydrolysis by intestinal enzymes, they can be fermented by the intestinal microflora [39]. By oral administration, these molecules have different biological activities related to the host’s well-being. 

Galacto-oligosaccharide ingestion modifies some features of the intestinal mucosa in mice. For example, galacto-oligosaccharides increase the mucosa-associated mucin content and enterocyte-associated sucrase activity in the small intestine without modifying villi heights [56]. 

Moreover, such saccharides are able to modulate intestinal barrier function [57] through direct stimulation of intestinal goblet cells [58]. The same authors indicated that this molecule can directly modulate the expression of some goblet cells’ secretory products (trefoil factor 3 (TFF3)) that support epithelial restitution and mucosal protection, and a Golgi sulfotransferase that contributes to the production of barrier-enhancing sulfomucins. The anti-allergic effects of this kind of oligosaccharide have been verified in mice. For instance, mice with atopic dermatitis exhibited fewer symptoms of dermatitis, showed higher levels of helper T cell 1 (Th1) cytokines and unchanged levels of helper T cell 2 (Th2) cytokines such as interleukin 13 (IL-13), enhanced IL-10, and inhibited IL-1β, IL-6, IL-17, and Tumor Necrosis Factor α (TNFα) under the oral administration of such molecules [59]. Moreover, galacto-oligosaccharides in milk formula have a positive effect on mucosal immunity [60]. Gourbeyre et al. [61] reported that such saccharides and inulin prebiotic mixture supplied in prenatal and post-weaning periods can differentially improve the immune response in mice towards a mechanism to prevents various immune pathologies such as allergies, autoimmune diseases, and inflammatory bowel disease [61].

Furthermore, many researchers have shown that galacto-oligosaccharides are able to reach the large intestine and can be metabolized by the indigenous microflora. They are structurally similar to cell surface glycoconjugates (adherent sites for pathogens) in the gastrointestinal tract [62]. Therefore, they can protect against bacterial colonization and invasion by pathogens and enhance the growth or activity of some health-promoting microflora [39]. Ben et al. [63] said that galacto-oligosaccharides (0.24 g/100 mL) in infant formula can improve stool frequency, decrease fecal pH, and stimulate intestinal *Bifidobacteria* and *Lactobacilli* populations.

In general, these molecules also confer benefits upon the mucosa and host health by improving mucosal structure and function, protecting the integrity of intestinal structure and function, modulating immune functions, and changing the gut’s microbial composition and related activities.

### 3.2. Fructo-Oligosaccharide

Fructo-oligosaccharide is extracted from the blue agave plant, fruits (such as bananas), vegetables (such as onions, chicory root, garlic, asparagus, and leeks), and some grains and cereals (such as wheat and barley) [64]. This molecule also has some other functional characteristics such as altering actin filament distribution and increasing the intestinal barrier function [65]. Moreover, it improves the activities of total protease and amylase during oral supplementation [66]. 

Fructo-oligosaccharides can modulate various parameters of the immune system. Schley and Field [67] provided some evidence on the possible mechanisms of the immunomodulatory effects of fructo-oligosaccharide. For example, a dose of 30 g/L in drinking water or 10 g/day can increase gut-associated lymphoid cells (including CD4^+^, CD8^+^, T-cells, B-cells, macrophages, and eosinophils) [39]. 

Moreover, such molecules can be fermented by the intestinal microflora [68], reducing pH and providing the production of gases and acids. The latter provide some energy to the body. Fructo-oligosaccharides selectively stimulate the growth of probiotic-like bacteria that are part of the commensal gut microbiota. For example, fructo-oligosaccharides exert a preferential stimulatory effect on several bacteria of the health-promoting genus *Bifidobacterium* and *Lactobacillus*, while maintaining populations of unprofitable or potential pathogens (*E. coli*) at relatively low levels in the small intestine and cecal digesta [39].

Another interesting feature of fructo-oligosaccharides is their possible role in mineral absorption. Several studies showed that fructo-oligosaccharide can also promote calcium absorption in both animal and human guts [69]. There are two ways to explain the reason why fructo-oligosaccharide can affect the mineral absorption. One way is mainly related to the fermentation of fructo-oligosaccharide by resident microbiota [70]. Another way is associated with the extended mineral absorption area. 

Therefore, functional oligosaccharides can promote gut development, improve host’s well-being [11], and reduce the risk of lifestyle-related diseases (such as cardiovascular disease, cancer, and obesity) [12] by altering the composition of intestinal bacteria, improving the balance of intestinal microflora, stimulating the growth of health-promoting bacteria [9], decreasing gastrointestinal infections [71], and modifying intestinal fermentation processes.

### 3.3. Mannan-Oligosaccharide

Mannan-oligosaccharide is derived from the cell wall of yeast (*Saccharomyces cerevisiae*). It has several biological functions related to the host’s health. Application of a mannan-oligosaccharide diet leads to increased body weight in poultry and swine [72]. Mannan-oligosaccharide can also improve weight gain, feed conversion [73], and productivity in carp and *Cyprinus carpio* var. Jian [74]. Staykov et al. [75] have shown that mannan-oligosaccharide improves growth performance. Moreover, mannan-oligosaccharide–protein conjugates are involved in interactions with the animal’s immune system [76] and reduces the mortality rate of juvenile carp [77] and rainbow trout [75].

Furthermore, dietary mannan-oligosaccharide improves intestinal morphology and modulates the functions in the anterior and posterior regions of the intestines. It increases the absorptive surface area by promoting longer mucosal foldings. This oligosaccharide can increase microvilli density and length. Mannan-oligosaccharide can improve intestinal morphology and epithelial brush borders by modulating intestinal microbial communities [78]. As a natural nutritional supplement, mannan-oligosaccharide offers a novel approach to supporting the microflora and thus improving intestinal health. For example, Spring et al. [79] indicated that mannan-oligosaccharides reduce the prevalence and the concentration of different strains of *Salmonella*, as well as *E. coli* [79]. Baurhoo et al. [80] reported that mannan-oligosaccharide promotes beneficial bacteria such as *Lactobacilli* and *Bifidobacteria*. 

The improvement in digestive functions is related to the structure of mannan-oligosaccharides, which allows it to bind to various receptors in animal digestive tracts [81] and to the receptors on bacterial membranes [82]. The binding of mannose to the intestinal tract improves animal general health and reduces the risk of pathogen colonization.

Therefore, mannan-oligosaccharide has some effects on growth performance, immune modulation, intestinal health, and microbial composition of the host.

### 3.4. Chitosan Oligosaccharides

Chitosan oligosaccharides, a degradation product of chitosan, are derived from chitin from the exoskeletons of shrimps, crabs, and insects. Chitosan oligosaccharides can be absorbed by intestinal epithelial cells without digestion by gastrointestinal enzymes or gut flora [39]. Chitosan has been applied as an animal dietary supplement and a replacement for feed-grade antibiotics as it possesses special physicochemical properties (such as water solubility, low viscosity, intestinal absorbability, and bioactivity) and various biological functions. 

Muanprasat et al. [83] revealed that chitosan promotes growth performance and improves intestinal health. Chitosan oligosaccharide supplementation improves performance, nutrient digestibility, serum composition, and microbial ecology in weanling pigs [84] and broiler chickens [77]. The improved growth performance from chitosan oligosaccharides may be related to an increase in feed intake [85], nutrient digestibility [86], and serum growth hormone (GH) and insulin-like growth factor 1 (IGF-I) concentrations [85]. Dietary supplementation with chitosan at 200 mg/kg increases villi heights and the villus:crypt ratio in the jejunum and ileum of weanling pigs [87]. Chitosan oligosaccharides at 400 or 600 mg/kg improves gut barrier function [39]. 

Moreover, some immunological roles have been established for chitosan such as anti-inflammatory, anti-oxidative, and antibacterial activities [39]. Yousef et al. [88] reported that chitosan can alleviate inflammation and inflammatory bowel disease by suppressing the responses of nuclear factor kappa B (NF-κB)-mediated inflammation in intestinal epithelial cells. 

Chitosan oligosaccharides can also stimulate the populations of beneficial flora such as *Bifidobacteria* and *Lactobacilli* and decrease harmful flora such as *S. aureus* in the cecum of weanling pigs [39]. Another report said that dietary supplementation with chitosan oligosaccharides increases the population of *Lactobacilli* and decreases the counts of *E. coli* in the feces of weaned pigs [89]. One possible explanation for chitosan’s antimicrobial activity is that the positive charge on the NH^3+^ group of the glucosamine monomer of such molecules allows interactions with negatively charged microbial cell membranes that leads to the leakage of intracellular constituents [90]. 

The regulation of intestinal functions, modification of small-intestinal mucosal architecture, immune system, and modulation of the microbial habitat have been recognized as vital ways to improve growth performance of weaned pigs by chitosan [84]. These trials indicated that chitosan improves growth performance, the intestinal structure, barrier functions, and generally the host’s state of well-being at different ages. 

### 3.5. Cello-Oligosaccharide

Cello-oligosaccharide is a functional oligosaccharide, which is produced from cellulose degradation [91] in wheat straw, rice husk, and *Medicago sativa* [92]. This oligosaccharide has different bioactive patterns. For instance, the addition of this molecule to pig diets influences intestinal architecture by increasing villi heights, villus height/crypt depth ratio, and villi surface area. The increased height of the villi leads to an increased digestive and absorptive function of the intestine due to an increased absorptive surface area, expression of brush border enzymes, and nutrient transport systems [93]. 

In addition, cello-oligosaccharide supplementation increases intestinal nutrient transport such as sodium-dependent glucose transport and l-glutamine transport [94]. Leeson et al. [95] indicated that cello-oligosaccharide can be hydrolyzed to short-chain fatty acids, such as butyric acid, in the gastrointestinal tract. Such hydrolysis products can be described as a source of energy for enteric mucosa and a stimulant of villi growth.

Incremental cello-oligosaccharide effectively improves intestinal microflora, morphology, and barrier integrity in broilers subjected to heat stress, indicating that the adverse effects caused by heat stress on intestinal microflora and structure can be relieved by cello-oligosaccharide supplementation [96]. Jiao et al. [94] reported that cello-oligosaccharide supplementation in weaned pigs increases the population of *Lactobacillus* and decreases *Clostridium*.

Thus, cello-oligosaccharide can provide multiple positive functions toward the host’s health, intestinal mucosal architecture, absorption function, barrier integrity, and gut’s microflora.

The functions of different functional oligosaccharides on the intestinal tract are summarized in Table 1.

## 4. Relationship between SBA and Oligosaccharides

Based on the structural characteristic of SBA, it specifically binds *N*-glyphthalide-d-galactosamine or galactose. X-ray crystal structures of SBA confirm the oligomeric structure [97] and that each subunit of SBA has a covalently linked oligosaccharide chain. Many of the branch-chain oligosaccharides have been shown to be multivalent and to bind, cross-link, and precipitate with specific multivalent lectins [98]. Such interactions can lead to the formation of homogeneous cross-linked complexes between SBA and oligosaccharides and glycopeptides [99]. These complexes between SBA and synthetic multiantennary oligosaccharides (with terminal Gal or GalNAc residues) include a series of blood group I antigen analogs [98]. The unique cross-linked lattices between four isomeric biantennary oligosaccharides and SBA have been tested by X-ray crystallographic studies [100]. Ujita et al. [101] suggested SBA binds to oligosaccharides and decreases during sialylation (the process by which sialic acid groups are introduced onto molecules such as oligosaccharides and carbohydrates as the terminal monosaccharide). Thus, SBA has the activity of a multivalent carbohydrate ligand. Despite the above indications, there is no data that supports the binding of non-digestible dietary oligosaccharides to the agglutinin to block its actions. For this to occur, the affinity of these oligosaccharides must be greater than the affinity of the agglutinin for its binding sites on intestinal epithelial cells.

The specific sugar binding of SBA can inhibit the biological activity of SBA, which reduces the combination of SBA with the intestinal epithelial cells and reduces the damage to the structure and function of the intestinal tract. SBA preferentially binds to oligosaccharides and the binding can be blocked by substitutions on the penultimate sugars (binding site). For example, an injection of SBA can exhibit the harmful effect on the small intestine and induce a typical inflammatory response. This hazardous effect can be blocked by pretreatment with *N*-acetylgalactosamine or mannose but not glucose or fructose [38]. These data strongly indicate that neutrophil accumulation is induced by SBA depending on its specific carbohydrate-binding properties. One reason explaining the role of *N*-acetylgalactosamine or mannose on the inhibition of an SBA-induced inflammatory response is that SBA is a glycoprotein containing *N*-acetylgalactosamine and mannose in its structure [102]. In addition, galactose can stabilize the secondary structure of SBA [18]. However, there are differences in the degree of SBA binding to different oligosaccharides. For example, the lactose-binding capacity of SBA is 100 times lower than for the oligosaccharide Gal-Naccll-3Gal [103]. The specific combination of SBA with intestinal epithelial cells is the precondition for its anti-nutritional functions [104]. Therefore, from a structural view, we can consider applying some oligosaccharides to reduce the SBA anti-nutritional effects by blocking the structural activities of SBA. However, more research is needed to demonstrate the blocking effect of some other functional oligosaccharides on SBA.

Apart from the close structural relationship between SBA and oligosaccharides, they also have some relations in their functions. SBA and oligosaccharides have opposite effects on intestinal structure and function. SBA mediates negative effects on animal intestinal health by damaging the intestinal structure and barrier function, inducing inflammation, and causing an imbalance in the intestinal flora. On the contrary, functional oligosaccharides are beneficial to the host’s health by improving mucosal structure and function, protecting the integrity of the intestinal structure, modulating the immunity, and balancing the gastrointestinal microbiota. Therefore, functional oligosaccharides can be used to alleviate the negative effects of SBA on the structure and function of the animal’s intestines.

Maenz et al. [25] indicated that some lactic acid bacteria can diminish the cytotoxic effects of SBA in mice IECs by binding the SBA molecules to carbohydrates on the bacterial surface. Babot et al. [105] concluded that the early administration of *Bifidobacterium infantis* CRL1395, containing a strain expressing *N*-acetyl-d-galactosamine on its surface can effectively reduce the toxicity of SBA. Functional oligosaccharides can act as a regulator of the growth of *Bifidobacterium*. 

Regarding rumen fermentation in ruminants that reduce the activity of the anti-nutritional factors, the anti-nutritional effect of SBA in monogastric animals is greater than that in ruminants [7]. Monogastrics contain no endogenous enzymes that are capable of digesting certain oligosaccharides such as raffinose and stachyose [49]. Therefore, the use of functional oligosaccharides to alleviate the anti-nutritional effects of SBA is mainly suitable for monogastric animals.

**Figure 2:**
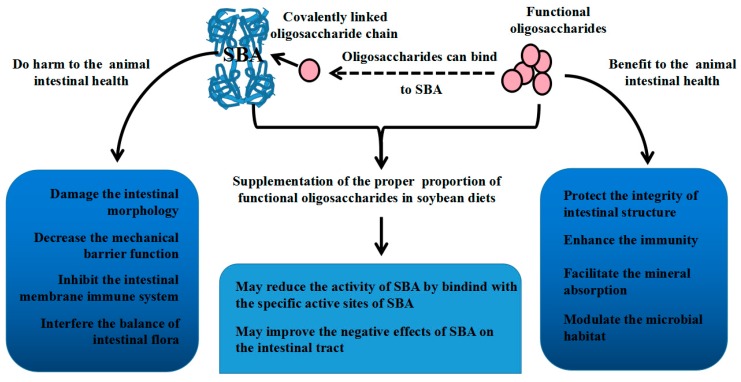
The relationship between SBA and oligosaccharides.

The relationships between SBA and oligosaccharides are shown in Figure 2.

## 5. Conclusions

SBA negatively affects intestinal structure, intestinal permeability, mucosal immune system, and intestinal flora. Such implications could reduce feed utilization and growth, and could cause disease and even death. In contrast, functional oligosaccharides have a positive effect on intestinal structure and function, barrier functions, intestinal immunity, and microbial community balance. In addition, according to the characteristics of SBA and functional oligosaccharides, neither can be digested by digestive enzymes. Therefore, supplementation with the proper proportion of functional oligosaccharides in soybean diets may improve the negative effects of SBA on the intestinal tract. Besides, according to the function of different functional oligosaccharides, some oligosaccharide combinations can be selected to alleviate the intestinal structural and functional damage caused by SBA.

## Figures and Tables

**Table 1:** The main functions of different functional oligosaccharides on the intestinal tract.

Types of Functional Oligosaccharide	Growth Performance	Intestinal Structure and Function	Immune System	Intestinal Microflora	References
Galacto-oligosaccharide		Increase the content of small intestinal mucosa-associated mucin and enterocyte-associated sucrase activity without modifying villi heights Modulate intestinal barrier function	Have a positive effect on mucosal immunity Prevents various immune pathologies such as allergies, autoimmune diseases, and inflammatory bowel disease	Protect against bacterial colonization and invasion by pathogens Enhance the growth or activity of some health-promoting microflora	[55,56,57,58,59,60,61,62,63]
Fructo-oligosaccharide		Alter actin filament distribution Increase the intestinal barrier function Improve the activities of total protease and amylase	Modulate various parameters of the immune system Increase gut-associated lymphoid cells (including CD4^+^, CD8^+^, T-cells, B-cells, macrophages, and eosinophils)	Reduce pH Provide the production of gases and acids Stimulate the growth of probiotic-like bacteria	[64,65,66,67,68,69,70,71]
Mannan-oligosaccharide	Increase body weight Improve weight gain and feed conversion	Improve intestinal morphology Modulate functions in the anterior and posterior intestinal regions Increase absorptive surface area Increase microvilli density and length		Offer a novel approach to support the microflora	[72,73,74,75,76,77,78,79,80,81,82]
Chitosan oligosaccharide	Improve growth performance, nutrient digestibility, feed intake, serum composition, and microbial ecology	Increase villi heights and villus:crypt ratio Improve gut barrier function	Have anti-inflammatory, anti-oxidative, and antibacterial activities	Stimulate the populations of beneficial flora Decrease the number of harmful flora	[83,84,85,86,87,88,89,90]
Cello-oligosaccharide		Increase villi heights, villus height/crypt depth ratio, and villus surface area Increase the expression of brush border enzymes Increase nutrient transport systems Improve the barrier integrity		Improve the intestinal microflora	[91,92,93,94,95,96]
